# Bio-Inspired Ghost Imaging: A Self-Attention Approach for Scattering-Robust Remote Sensing

**DOI:** 10.3390/biomimetics11010053

**Published:** 2026-01-08

**Authors:** Rehmat Iqbal, Yanfeng Song, Kiran Zahoor, Loulou Deng, Dapeng Tian, Yutang Wang, Peng Wang, Jie Cao

**Affiliations:** 1 Key Laboratory of Biomimetic Robots and Systems, Ministry of Education, Beijing 100081, China; 3820212008@bit.edu.cn; 2 School of Optics and Photonics, Beijing Institute of Technology, Beijing 100081, China; 3 Bionic Robots and Photonics Key Laboratory, Ministry of Education, School of Optics and Photonics, Beijing Institute of Technology, Beijing 100081, China; 4 Xi’an Moder Control Technology Research Institute, Xi’an 710018, China; 5 School of Mathematics and Statistics, Beijing Institute of Technology, Beijing 100081, China; 6 College of Optical Science and Engineering, Zhejiang University, Hangzhou 310027, China; 7 State Key Laboratory of Dynamic Optical Imaging and Measurement, Changchun Institute of Optics, Fine Mechanics and Physics, Chinese Academy of Sciences, Changchun 130033, China; 8 University of Chinese Academy of Sciences, Beijing 100049, China; 9 Beijing Aerospace Measurement and Control Technology Co., Ltd., Beijing 100041, China; 10 National Key Laboratory on Near Surface Detection, Beijing 100072, China

**Keywords:** ghost imaging, remote sensing, scattering, self-attention, transformer, fog, bio-inspired

## Abstract

Ghost imaging (GI) offers a robust framework for remote sensing under degraded visibility conditions. However, atmospheric scattering in phenomena such as fog introduces significant noise and signal attenuation, thereby limiting its efficacy. Inspired by the selective attention mechanisms of biological visual systems, this study introduces a novel deep learning (DL) architecture that embeds a self-attention mechanism to enhance GI reconstruction in foggy environments. The proposed approach mimics neural processes by modeling both local and global dependencies within one-dimensional bucket measurements, enabling superior recovery of image details and structural coherence even at reduced sampling rates. Extensive simulations on the Modified National Institute of Standards and Technology (MNIST) and a custom Human-Horse dataset demonstrate that our bio-inspired model outperforms conventional GI and convolutional neural network-based methods. Specifically, it achieves Peak Signal-to-Noise Ratio (PSNR) values between 24.5–25.5 dB/m and Structural Similarity Index Measure (SSIM) values of approximately 0.8 under high scattering conditions (β ≥ 3.0 dB/m) and moderate sampling ratios (N ≥ 50%). A comparative analysis confirms the critical role of the self-attention module, providing high-quality image reconstruction over baseline techniques. The model also maintains computational efficiency, with inference times under 0.12 s, supporting real-time applications. This work establishes a new benchmark for bio-inspired computational imaging, with significant potential for environmental monitoring, autonomous navigation and defense systems operating in adverse weather.

## 1. Introduction

Ghost imaging (GI) has emerged as a powerful computational imaging technique with significant potential for remote sensing applications, offering innovative solutions to constraints inherent in conventional imaging systems [1]. By leveraging the correlation between spatially structured illumination and a single-pixel detector (SPD), GI provides notable robustness to noise and reduced hardware complexity. However, its performance is critically degraded in scattering media, such as fog, haze, and turbulence, where image reconstruction suffers from severe contrast loss, blurred edges, and diminished detail fidelity [2]. These challenges are particularly acute in real-time imaging, where stringent constraints on measurement time and sampling rates further impair practical utility.

To confront the challenge of imaging through obscurants, we can look to biological systems that have evolved advanced strategies for perception in degraded visual environments. From the neural circuitry in the mammalian visual cortex that performs selective noise suppression to the ability of certain marine species to discern structures in turbid water, biology demonstrates the power of attention-based processing to prioritize salient information from noisy sensory inputs [3,4,5,6]. This bio-inspired principle, which focuses computational resources on the most informative features, offers a compelling paradigm for advancing computational imaging under adverse conditions.

Driven by this insight, our work introduces a bio-inspired deep learning (DL) framework for GI that embeds a self-attention mechanism to mimic selective processing. Traditional techniques for improving GI, such as iterative denoising [7], scalar matrix methods [8], differential GI [9], and Hadamard-based approaches [10], are fundamentally based on linear correlation. Under high scattering conditions, the bucket signal becomes dominated by noise, causing these methods to fail as the underlying correlation assumption breaks down. While DL, particularly Convolutional Neural Networks (CNNs), has improved GI [11,12], their localized receptive fields struggle to model the long-range dependencies necessary to distinguish a coherent global structure from globally pervasive scattering noise. Our bio-inspired transformer architecture addresses this directly by using a self-attention mechanism to integrate global context, learning a nonlinear mapping from degraded bucket signals to clear images that is inherently robust to this noise [13].

The advent of Transformer-based architectures, with their core Multi-Head Self-Attention (MHSA) mechanism [14], presents a transformative opportunity to capture these global contextual relationships, as initially demonstrated in vision tasks by the Vision Transformer (ViT) [14]. However, the direct application of standard Transformers to GI in low-visibility conditions is hampered by their prohibitive computational cost. Although efficient variants like SNR-Net [15] and Uformer [16] have been proposed to mitigate this through architectural optimizations, the potential of tailored attention mechanisms to simultaneously reduce sampling requirements, improve reconstruction fidelity, and maintain efficiency for GI in fog remains largely untapped.

The main objective of this study are:

- To develop a novel, bio-inspired DL architecture incorporating a self-attention mechanism for robust GI reconstruction technique in foggy environments for remote sensing applications.
- To enable high-fidelity image recovery from highly compressed and severely degraded bucket signals.
- To rigorously evaluate the model performance against state-of-the-art methods and through an ablation study to quantify the contribution of the self-attention mechanism.
- To demonstrate the hybrid model computational efficiency, ensuring its viability for real-time remote sensing applications.

To overcome this gap by developing a bio-inspired self-attention architecture for GI, explicitly designed for a spectrum of fog density. This work introduces a novel, bio-inspired architecture that adapts self-attention to the unique challenge of reconstructing images from globally degraded, one-dimensional bucket signals. Our model is specifically designed to capture the long-range dependencies necessary to overcome scattering noise, a capability that localized convolutional filters lack. We rigorously train and validate our model on the MNIST and a remote sensing Human-Horse dataset under varying scattering coefficients (β∈{0.1, 0.5, 1.0, 2.0, 3.0, 5.0} dB/m) and sampling ratios (N∈{10%, 25%, 50%, 75%, 100%}). The results demonstrate that our method achieves superior reconstruction quality, with PSNR values up to 24.5–25.5 dB and SSIM values of approximately 0.8–0.85 under high-fog conditions (β ≥ 3.0 dB/m) and moderate sampling ratios (N ≥ 50%), underscoring its significant potential for applications in environmental monitoring, security surveillance, and defense.

## 2. Proposed Model and Prior

### 2.1. Ghost Imaging in Foggy Environments

Building upon the bio-inspired premise of our approach, this section details the computational imaging framework designed to emulate the robustness of biological vision in scattering media. The core challenge in foggy environments is the severe degradation of optical signals, a problem that natural systems overcome through efficient neural processing. Our method addresses this by augmenting the physical GI setup with a neural-inspired reconstruction network that mimics this selective, attention-driven processing.

**Figure 1:**
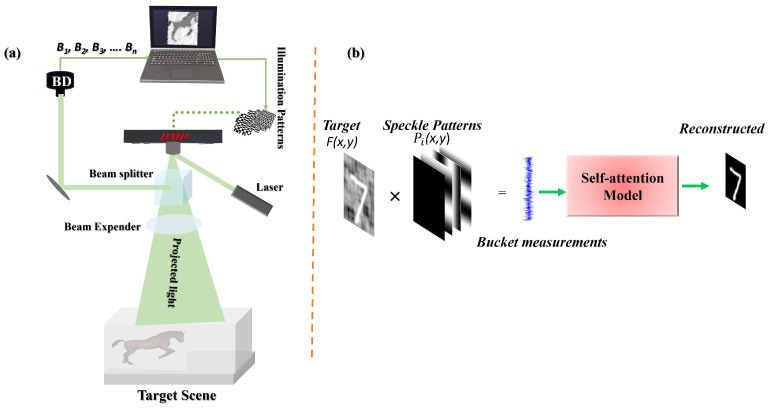
(**a**) Schematic of the GI system operating in a foggy medium. A laser beam is modulated by a DMD to generate structured patterns, which are directed through a BS and BE for long-range propagation. The light scattered by the object is captured by a bucket detector (BD). (**b**) The computed bucket signals are processed by a self-attention based DL model to generate a high-quality image, emulating a neural-inspired reconstruction pathway.

The standard GI process begins with a series of structured illumination patterns $P_{i}\left(x,y\right)$ preloaded onto a Digital Micromirror Device (DMD) and speckle patterns were generated according to Equation (3) with N=64 for the 64×64 Human-Horse dataset and N=28 for the MNIST dataset. The distance between the DMD and the target object was simulated as d=50  m. As illustrated in Figure 1a, a laser illuminates the DMD, and the modulated light is directed through a beam splitter (BS) and a beam expander (BE) to ensure sufficient spatial coverage for long-range propagation. This structured light then traverses the foggy medium S(x,y), undergoing significant scattering and distortion before illuminating the target object F(x,y). A SPD collects the resulting light, which has interacted with both the object and the scattering medium, producing a one-dimensional bucket measurement vector $H_{m}$. In foggy environments, the scattering medium S(x,y) severely degrades the optical signal through two primary mechanisms: attenuation and additive scattering noise. Attenuation reduces the intensity of the light incident on the target F(x,y), weakening the object’s reflected signal and leading to a lower signal-to-noise ratio (SNR) in the bucket measurement $H_{m}$. Additive noise arises from photons being scattered by fog particles directly into the bucket detector without interacting with the target. This introduces uncorrelated noise into the bucket signal. In the conventional correlation reconstruction Equation (1), this combined effect manifests as a significant loss of contrast, blurred edges, and a noisy, low-fidelity image $O_{GI}$ as the correlated signal from the object is effected by the uncorrelated scattering noise.

The conventional correlation-based reconstruction in GI is expressed as:

(1) $$\begin{array}{c} O_{GI}=\frac{1}{n}\sum_{i=1}^{n}\left(H-\langle H\rangle \right)P_{i}\left(x,y\right), \end{array}$$

where *H* is the bucket measurement vector. The value of each bucket measurement $H_{i}$ is given by:

(2) $$\begin{array}{c} H_{i}=\int P_{i}\left(x,y\right)\times F\left(x,y\right)\times S_{i}\left(x,y\right)\;dxdy, \end{array}$$

**Figure 2:**
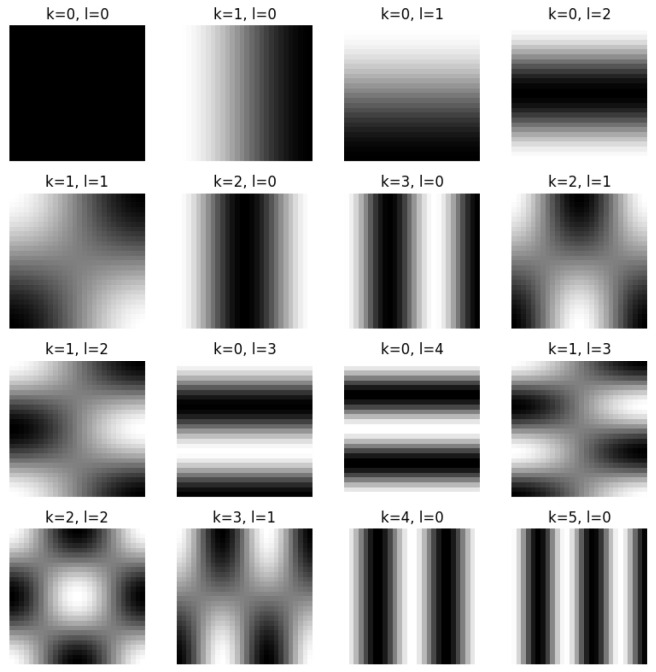
Illustration of illumination patterns utilized in GI under scattering environment.

Here, $P_{i}\left(x,y\right)$ represents the *i*-th illumination pattern as illustrated in Figure 2, with i=1,2,…,M, and *M* being the total number of measurements. In this work, we employ speckle patterns formulated as:

(3) $$P_{k,l}\left(x,y\right)=\alpha_{k}\alpha_{l}\cos\left(\frac{\pi k(2x+1)}{2N}\right)\cos\left(\frac{\pi l(2y+1)}{2N}\right),$$

where,

$$\alpha_{k}=\left\{\begin{array}{cc} \sqrt{\frac{1}{N}} & \mathrm{if}\;k=0\\ \sqrt{\frac{2}{N}} & \mathrm{if}\;k\neq 0 \end{array}\right.,\;\alpha_{l}=\left\{\begin{array}{cc} \sqrt{\frac{1}{N}} & \mathrm{if}\;l=0\\ \sqrt{\frac{2}{N}} & \mathrm{if}\;l\neq 0. \end{array}\right.$$

While this physical setup provides the raw data, the conventional reconstruction in Equation (1) falters under strong scattering, much like a rudimentary sensory system without higher-level processing. To overcome this, we introduce a neural-inspired reconstruction model that learns a direct, intelligent mapping from the degraded 1D bucket signals to a clear 2D image. This process, which replaces the linear correlation of standard GI, is described as:

(4) $$\hat{I}\left(x,y\right)=R\left(H\right),$$

where $H={\left[H_{1},H_{2},\ldots ,H_{M}\right]}^{T}$ is the vector of bucket measurements (the correlation of reflected light and known illumination patterns), and R(·) is the complex, nonlinear reconstruction function learned by our self-attention model. The output $\hat{I}\left(x,y\right)$ approximates the true reflectivity distribution of the object.

**Figure 3:**
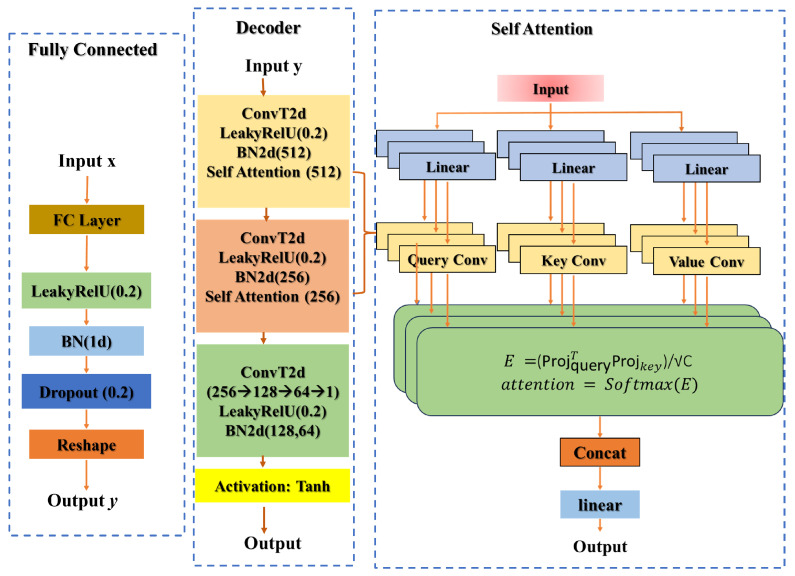
Schematic illustration of the proposed encoder-decoder network for reconstructing high-quality 2D images from compressed 1D bucket measurements under foggy conditions. The model integrates a specialized self-attention mechanism within the decoder to capture long-range spatial dependencies, emulating the selective attention processes of biological visual systems.

The model is trained and evaluated using a synthetic pairs of foggy bucket signals and their corresponding ground truth clear images C(x,y). The architecture of our proposed bio-inspired attention-based Mechanism for Foggy-condition GI Reconstruction (AMFR-GI) is shown in Figure 3. The main part is a self-attention mechanism, bio-inspired by the brain’s ability to focus on salient features and suppress noise. This allows the model to capture long-range dependencies within the compressed measurement data, effectively “attending” to the most informative components to achieve robust image recovery under various fog density and sampling rates, thereby realizing a computational form of selective perceptual attention.

### 2.2. Self-Attention Mechanism

The self-attention mechanism is bio-inspired by the brain’s capacity for selective attention, which allows biological visual systems, feedback connections modulate neural activity to prioritize salient features while suppressing noise, a process essential for perceiving objects in fog. Our artificial module functionally mimics this by dynamically calculating attention scores (Equations (6) and (7)), effectively working as a top-down attentional signal that highlights informative features in the bucket signal and suppresses noise, thereby enabling the network to ‘see through’ scattering media such as fog. In our model, this is implemented computationally. The mechanism allows the network to dynamically weight the importance of all elements in the compressed measurement sequence. When processing a fog degraded bucket signal, the model learns to ‘attend to’ or prioritize components that correspond to the true object’s structure while suppressing components correlated with scattering noise. This global, content-aware feature extraction is enables the accurate recovery of structural coherence and fine details that are lost in conventional methods. The architecture comprises two components: the self-attention module, which performs feature refinement through neural-inspired attention, and the self-attention reconstructor, which progressively builds the image from compressed measurements.

#### 2.2.1. Self-Attention Module

The self-attention module functions as an artificial neural circuit that learns complex spatial relationships across different regions of the input feature map. Bio-inspired by the top-down modulation observed in biological visual systems, this module dynamically weights the importance of different features, effectively focusing computational resources on the most informative elements while suppressing noise, a critical capability for imaging in scattering environments.

The module transforms an input feature map $x\in R^{B\times C\times H\times W}$ (where *B* denotes batch size, *C* channels, and H×W spatial dimensions) through three parallel 1×1 convolutional layers to extract query (*Q*), key (*K*), and value (*V*) projections:

(5) $$\begin{array}{cc} {\mathrm{proj}}_{\mathrm{query}} & ={\mathrm{Conv}}_{\mathrm{query}}\left(x\right)\\ {\mathrm{proj}}_{\mathrm{key}} & ={\mathrm{Conv}}_{\mathrm{key}}\left(x\right)\\ {\mathrm{proj}}_{\mathrm{value}} & ={\mathrm{Conv}}_{\mathrm{value}}\left(x\right) \end{array}$$

These projections undergo spatial reshaping to facilitate the computation of attention scores, which determine how much focus to place on different parts of the input:

(6) $$E=\frac{{\mathrm{proj}}_{\mathrm{query}}^{⊤}{\mathrm{proj}}_{\mathrm{key}}}{\sqrt{C}}$$

where $E\in R^{HW\times HW}$ represents the attention energy matrix, and $\sqrt{C}$ provides scaling stabilization in high-dimensional spaces.

The attention probabilities are obtained through softmax normalization:

(7) attention=Softmax(E)

The final attended features are computed as a weighted combination of value projections:

(8) $$\mathrm{out}={\mathrm{proj}}_{\mathrm{value}}\cdot {\mathrm{attention}}^{⊤}$$

A residual connection with learnable scaling preserves gradient flow and enables adaptive feature integration:

(9) $${\mathrm{out}}_{\mathrm{final}}=\gamma \cdot \mathrm{out}+x$$

where γ∈R is a trainable parameter initialized to zero, allowing the network to progressively learn the optimal contribution of attention. This design enables selective focus on relevant spatial locations while maintaining original feature information, effectively mimicking the brain’s ability to enhance salient features in noisy sensory input.

#### 2.2.2. Self-Attention Reconstructor

The SelfAttentionReconstructor forms the image formation pathway of our bio-inspired architecture, transforming compressed GI measurements into high-fidelity images through a hierarchical decoding process. Following an encoder-decoder paradigm, this component maps the input measurement vector $x\in R^{B\times N}$ to a compact latent representation:

(10) $$x_{\mathrm{latent}}=\mathrm{FC}\left(x\right)$$

The latent representation is reshaped into a 4D tensor (B,1024,8,8) and progressively decoded through transposed convolutional layers that systematically increase spatial resolution while integrating self-attention modules at multiple scales:

(11) $$x_{\mathrm{reconstructed}}=\mathrm{Decoder}\left(x_{\mathrm{latent}}\right)$$

The decoder architecture employs:

(a) A cascade of transposed convolutional layers with batch normalization and Leaky ReLU activations, interspersed with self-attention modules to capture multi-scale contextual relationships;
(b) A final convolutional layer with hyperbolic tangent activation producing normalized output in [−1,1].

The complete reconstruction process is defined as:

(12) $$\hat{I}=\mathrm{SelfAttentionReconstructor}\left(M\right)$$

where $\hat{I}\in R^{B\times 1\times H\times W}$ represents the reconstructed image and $M\in R^{B\times N}$ the input measurements. This formulation enables the transformation of low-dimensional compressed signals into high-resolution images through the synergistic integration of attention-guided feature processing and progressive spatial upsampling.

### 2.3. Training and Evaluation Framework

#### 2.3.1. Dataset Preparation

**Figure 4:**
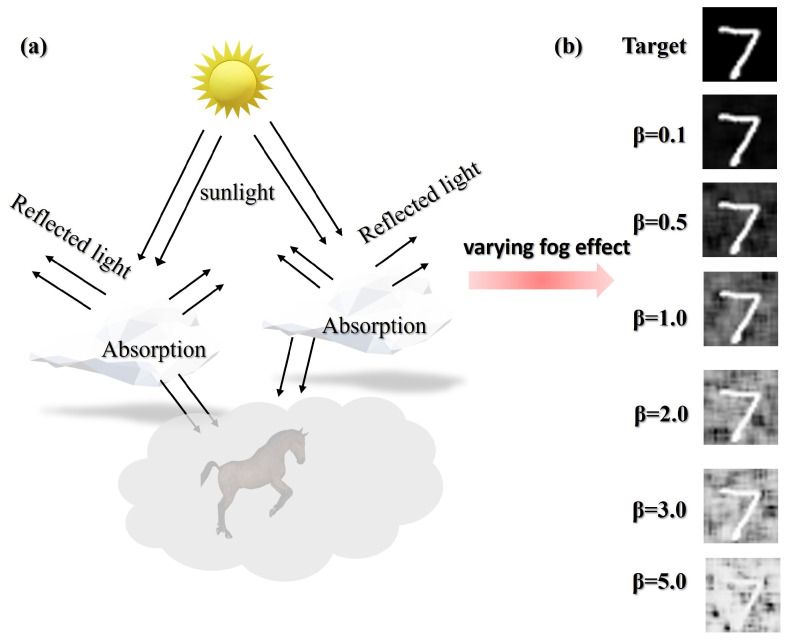
(**a**) Schematic diagram of atmospheric scattering model for simulating foggy environments (**b**) Visual impact of fog with varying scattering coefficient β.

To effectively train the bio-inspired AMFR-GI model, we generated a comprehensive dataset simulating realistic foggy conditions. Since acquiring paired clean and foggy ground-truth images is challenging, we developed a physical-based scattering model to synthesize fog effects on clear images I(x,y). The fog synthesis follows atmospheric scattering theory:

(13) $$\begin{array}{c} I_{f}\left(x,y\right)=I\left(x,y\right)\cdot \left(1-D\right)+F\left(x,y\right)\cdot D, \end{array}$$

where $I_{f}\left(x,y\right)$ is the fogged image, *D* represents fog density which was linearly scaled with the scattering coefficient β, and $F\left(x\right)=e^{-\beta d(x)}$ defines the fog transmission map with a constant distance d(x)=50 m across the scene to simulate uniform fog. The atmospheric scattering model is illustrated in Figure 4.

To enhance realism, we incorporated spatially varying fog with Gaussian noise:

(14) $$\begin{array}{c} F(x,y)=D\cdot N(0,\;1), \end{array}$$

followed by smoothing to introduce natural spatial coherence:

(15) $$\begin{array}{c} F_{s}=\frac{1}{K^{2}}\sum_{i=-\frac{K}{2}}^{\frac{K}{2}}\sum_{j=-\frac{K}{2}}^{\frac{K}{2}}F\left(x+i,y+j\right), \end{array}$$

The final fogged image is computed as:

(16) $$\begin{array}{c} I_{f}=\mathrm{clamp}\left(I\cdot \left(1-D\right)+F_{s},-1,1\right), \end{array}$$

preserving both scene content and realistic fog characteristics for training.

#### 2.3.2. Model Evaluation

To ensure a careful evaluation and prevent overfitting, the dataset was partitioned into three distinct subsets: training, validation, and testing. The training set was used to update the model parameters, the validation set was used for hyperparameter tuning and monitoring generalization performance during training, and the final performance metrics reported in Section 3 were computed on the held-out test set, which was never used in the training process.

The proposed model was implemented in PyTorch 2.1.1 and trained in a Python 3.12 environment on an NVIDIA RTX 3060 GPU. We employed Mean Squared Error (MSE) as the primary optimization objective:

(17) $$L_{\mathrm{MSE}}=\frac{1}{B\cdot H\cdot W}\sum_{i=1}^{B}\sum_{j=1}^{H}\sum_{k=1}^{W}{\left(I_{ijk}-{\hat{I}}_{ijk}\right)}^{2}.$$

The training procedure follows Algorithm 1. During each epoch, the model processes batches from the training set, computes the loss, and updates parameters via the Adam optimizer (learning rate $\eta =10^{-4}$, weight decay $10^{-5}$. Crucially, after processing the training batch, the model enters a validation phase where it evaluates the current state on the separate validation set without gradient computation. The Peak Signal-to-Noise Ratio (PSNR) and Structural Similarity Index (SSIM) are computed on this validation set for monitoring:

(18) $$\mathrm{PSNR}\left(I,\hat{I}\right)=10\cdot \log_{10}\left(\frac{{\mathrm{MAX}}_{I}^{2}}{\mathrm{MSE}(I,\hat{I})}\right),$$

and perceptual quality was assessed via Structural Similarity Index (SSIM):

(19) $$\mathrm{SSIM}\left(I,\hat{I}\right)=l\left(I,\hat{I}\right)\cdot c\left(I,\hat{I}\right)\cdot s\left(I,\hat{I}\right)$$

with component functions defined as:

(20) $$\begin{array}{cc} l(I,\hat{I}) & =\frac{2\mu_{I}\mu_{\hat{I}}+C_{1}}{\mu_{I}^{2}+\mu_{\hat{I}}^{2}+C_{1}} \end{array}$$

(21) $$\begin{array}{cc} c(I,\hat{I}) & =\frac{2\sigma_{I}\sigma_{\hat{I}}+C_{2}}{\sigma_{I}^{2}+\sigma_{\hat{I}}^{2}+C_{2}} \end{array}$$

(22) $$\begin{array}{cc} s(I,\hat{I}) & =\frac{\sigma_{I\hat{I}}+C_{3}}{\sigma_{I}\sigma_{\hat{I}}+C_{3}}, \end{array}$$

**Figure 5:**
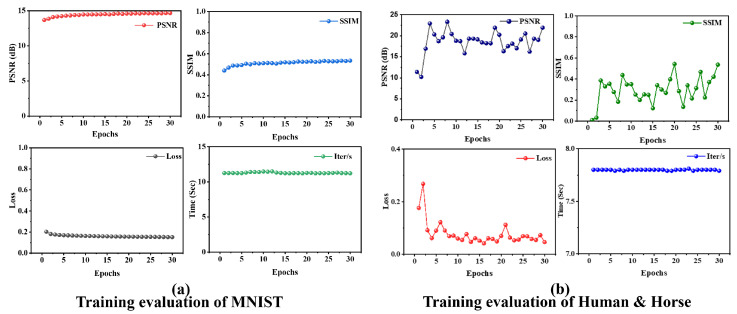
Training evaluation metrics for (**a**) MNIST (28 × 28 pixels) and (**b**) Human & Horse (64 × 64 pixels) datasets, demonstrating convergence and reconstruction performance across epochs.

Training and validation progress for both datasets is shown in Figure 5.
**Algorithm 1** Self-Attention Reconstruction Training with Validation
 **Require: **
Training dataset $D_{\mathrm{train}}$ with sampling ratio N∈[0,1], scattering coefficientβ∈[1dB/m,5dB/m] and fog density d∈[0,1]
 **Require: **
Validation dataset $D_{\mathrm{val}}$ for monitoring
 **Require: **
Model $f_{\theta }$ with parameters θ
 **Require: **
Learning rate η, batch size *B*, epochs *T*
 **Ensure: **
Trained model $f_{\theta }$  1:Initialize θ randomly  2:**for** epoch =1 to *T* **do**  3:    **Training Phase**  4:    **for** batch $\left(M,I\right)\in D_{\mathrm{train}}$ **do**  5:        $\hat{I}\leftarrow f_{\theta }\left(M\right)$                                                                                               ▹ Forward pass  6:        Compute $L_{\mathrm{MSE}}$ (Equation (17))                                                          ▹ Loss calculation  7:        Update θ via Adam optimizer                                                     ▹η, weight decay $10^{-5}$  8:    **end for**  9:    **Validation Phase**10:    Initialize accumulators: val_loss=0, val_psnr=0, val_ssim=0, count=011:    **for** batch $\left(M,I\right)\in D_{\mathrm{val}}$ **do**12:        $\hat{I}\leftarrow f_{\theta }\left(M\right)$                                                                            ▹ No gradient computation13:        Compute batch $L_{\mathrm{MSE}}$, PSNR (Equation (18)), SSIM (Equation (19))14:        Accumulate: $val\_loss\leftarrow val\_loss+L_{\mathrm{MSE}}$15:        Accumulate: val_psnr←val_psnr+PSNR16:        Accumulate: val_ssim←val_ssim+SSIM17:        count←count+118:    **end for**19:    Compute averages: val_loss=val_loss/count, val_psnr=val_psnr/count, val_ssim=val_ssim/count20:    Log validation metrics: val_loss, val_psnr, val_ssim for epoch monitoring21:**end for**22:**return** $f_{\theta }$

With the bio-inspired AMFR-GI architecture and comprehensive foggy dataset established, the model was systematically trained and validated across varying sampling ratios and fog density. The following section presents a detailed evaluation of the model’s performance under these challenging conditions.

## 3. Results

To validate the efficacy of our bio-inspired computational imaging framework, we conducted comprehensive numerical simulations under varying system parameters. The following analysis demonstrates how the self-attention mechanism modeled after neural selective attention enables robust image reconstruction in challenging foggy conditions across different sampling regimes and scattering intensities.

### 3.1. Reconstruction Performance Under Varying Sampling Ratios

This section investigates the performance of our bio-inspired GI model under varying sampling ratios, evaluating its ability to reconstruct images from sparse measurements in foggy environments. The scattering coefficient was fixed at β=1.0dB/m to simulate moderate fog conditions, while the sampling ratio *N* was varied across {10%,25%,50%,75%,100%} to assess data efficiency.

**Figure 6:**
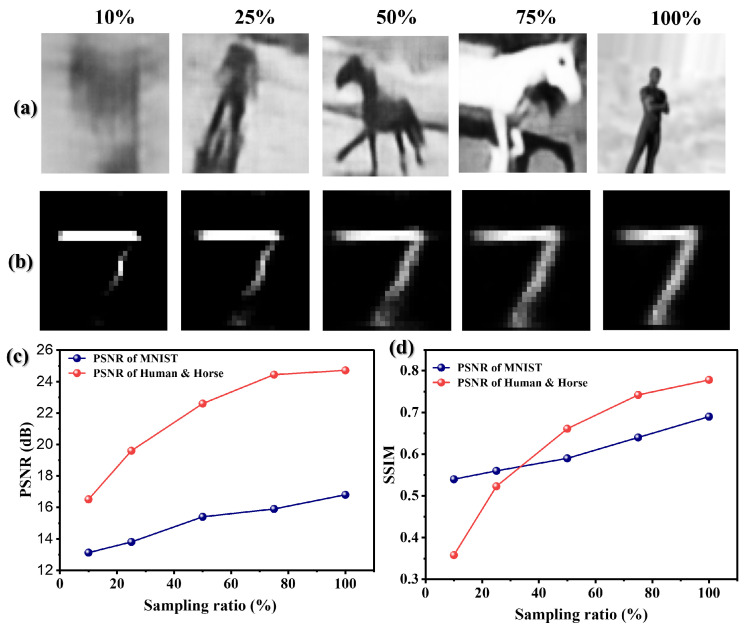
Performance evaluation under varying sampling ratios demonstrates the bio-inspired model’s data efficiency: (**a**) Human and Horse reconstructions show robust recovery even at 25% sampling, (**b**) MNIST reconstructions require higher sampling for complete recovery, (**c**) PSNR improvement with increased sampling, and (**d**) SSIM trends reflecting perceptual quality maintenance.

Figure 6 presents comprehensive qualitative and quantitative results for both the high-resolution Human and Horse dataset (64 × 64 pixels) and the low-resolution MNIST dataset (28 × 28 pixels). The bio-inspired architecture demonstrates remarkable capability in leveraging global contextual information emulating the brain’s ability to infer complete structures from partial data. At a sampling ratio of 25%, the Human and Horse dataset achieves near-complete reconstruction despite the scattering environment, with the self-attention mechanism effectively filling in missing spatial information. This mirrors biological vision systems that excel at perceiving coherent objects from fragmented visual cues in poor visibility.

Conversely, the MNIST dataset exhibits incomplete recovery at the same sampling ratio, reflecting the fundamental limitations of low-resolution data under scattering conditions. However, as sampling increases to 50%, both datasets achieve complete reconstruction, with the Human and Horse dataset maintaining superior visibility due to its richer structural information. The significant degradation observed at 10% sampling highlights the challenge of extreme undersampling in scattering media, where even sophisticated attention mechanisms struggle with insufficient signal content.

Quantitative metrics in Figure 6c,d reveal asymptotic improvement in both PSNR and SSIM with increasing sampling ratios, validating the model’s data-efficient learning capability. The Human and Horse dataset achieves PSNR ≈ 24.5 dB and SSIM ≈ 0.8 at higher sampling ratios, demonstrating robust reconstruction quality. The MNIST dataset shows lower baseline performance but significant improvement with increased sampling, indicating the model’s adaptive capability across resolution domains.

These findings underscore the advantage of our bio-inspired approach: by emulating the brain’s contextual processing, the model achieves substantial reconstruction quality at lower sampling ratios, enabling energy-efficient and time-constrained sensing systems for practical deployment in challenging environmental conditions.

### 3.2. Robustness to Progressive Scattering Effects

To evaluate the model’s resilience to increasingly adverse conditions, we systematically tested its performance under varying scattering coefficients while maintaining full sampling (N=100%). The scattering coefficient β was varied across {0.1dB/m,0.5dB/m,1.0dB/m,2.0dB/m,3.0dB/m,5.0dB/m} to emulate progressive fog density encountered in remote sensing applications.

**Figure 7:**
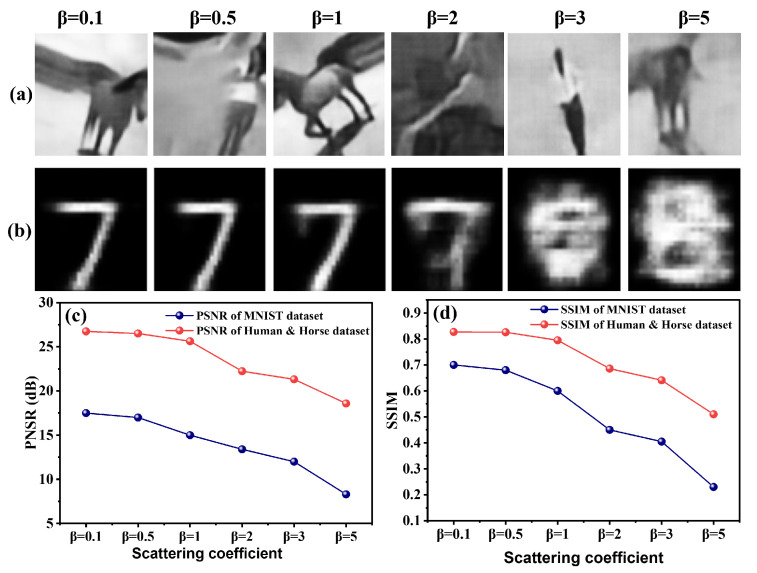
Bio-inspired model demonstrates scattering resilience: (**a**) Human and Horse reconstructions maintain structural coherence even at β=5.0dB/m, (**b**) MNIST shows progressive degradation with increasing scattering, (**c**) PSNR stability across scattering levels, and (**d**) SSIM preservation highlighting perceptual robustness.

Figure 7 presents the qualitative and quantitative results, revealing the model’s remarkable robustness to scattering-induced degradation. The bio-inspired attention mechanism demonstrates a capacity akin to biological vision in maintaining perceptual coherence despite signal deterioration. For the Human and Horse dataset (Figure 7a), reconstructed images maintain structural integrity and key features even at extreme scattering (β=5.0dB/m), with only gradual quality degradation. This resilience stems from the attention mechanism’s ability to prioritize and preserve semantically important features while suppressing noise, directly mirroring the neural processes that enable biological systems to see through fog and haze.

The MNIST dataset (Figure 7b) exhibits more pronounced degradation with increasing β, reflecting the inherent vulnerability of low-resolution, structurally simple images to scattering effects. The limited feature diversity in MNIST digits provides fewer robust cues for the attention mechanism to leverage under severe degradation, highlighting the interplay between image complexity and reconstruction robustness.

Quantitative analysis in Figure 7c,d confirms these observations: PSNR values show a general decline with increasing β, reflecting the fundamental signal-to-noise ratio challenge in dense fog. However, the Human and Horse dataset maintains significantly higher PSNR across all scattering levels, demonstrating the model’s superior handling of complex scenes. Similarly, the SSIM values show a progressive decline, but the high-resolution dataset maintains a relatively stable perceptual quality, whereas MNIST experiences a sharp drop, particularly beyond β=2.0dB/m.

This scattering resilience demonstrates the practical value of our bio-inspired approach for real-world remote sensing, where atmospheric conditions can vary dramatically. The model’s ability to maintain reconstruction quality across a wide scattering range positions it as a robust solution for environmental monitoring, security surveillance, and defense applications operating in degraded visual environments.

### 3.3. Comparative Analysis

**Figure 8:**
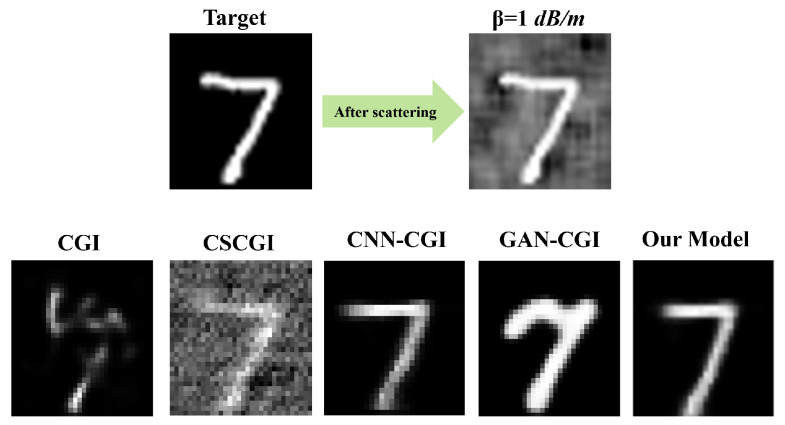
Comparative reconstruction performance under scattering conditions. The top row displays the target object from the (**left**) MNIST and (**right**) scattering effect object. The second row shows the reconstructions using CGI, CSCGI, CNN-CGI, and GAN-CGI methods. Visual comparison demonstrates that the proposed method effectively recovers structural details, enhances contrast, and suppresses scattering-induced noise, outperforming the traditional GI approach.

To objectively assess the performance of our proposed bio-inspired model (AMFR-GI), we conducted a qualitative and quantitative comparison against several established benchmark methods as illustrated in Figure 8 and Table 1, respectively. These included conventional computational GI (CGI), compressive sensing-based CGI (CSCGI), convolutional neural network-based CGI (CNN-CGI), and generative adversarial network-based CGI (GAN-CGI). The evaluation was performed under consistent scattering conditions β=1.0dB/m at full sampling (N=100%) using the MNIST dataset. As illustrated in Figure 8, visual inspection reveals that the proposed AMFR-GI method more effectively recovers structural details, enhances image contrast, and mitigates scattering-induced noise, demonstrating superior reconstruction quality compared to both traditional and DL-based GI approaches.

### 3.4. Computational Efficiency and Performance Analysis

The proposed self-attention-based framework for GI demonstrate significant advancements in reconstruction quality, particularly under adverse foggy conditions. However, its computational efficiency and performance on low-resolution datasets, such as MNIST (28 × 28 pixels), warrant detailed examination to ensure scalability and applicability across diverse imaging scenarios.

#### 3.4.1. Computational Efficiency of the Bio-Inspired Architecture

The self-attention mechanism, core to our bio-inspired architecture, introduces a computational overhead that scales with input resolution, a known trade-off for its superior capacity to model global context. To quantify this, we evaluated inference time and memory usage on an NVIDIA RTX 3060 GPU. As summarized in Table 2, the model requires approximately 0.12 s per inference for the high-resolution Human and Horse dataset (64 × 64 pixels) and only 0.08 s for the MNIST dataset (28 × 28 pixels), with same GPU memory usage. These metrics confirm that the model’s computational cost is well within the bounds for real-time remote sensing applications, as the inference time remains orders of magnitude below the typical 1-s threshold for such tasks [10]. The efficiency of this bio-inspired approach demonstrates that the benefits of global contextual processing can be achieved without prohibitive computational cost.

#### 3.4.2. Performance Analysis Across Resolutions

The model’s performance exhibits a strong dependency on input complexity, a characteristic shared with biological visual systems that excel at parsing rich scenes. On low-resolution, structurally simple datasets like MNIST, performance at very low sampling ratios (N=10%) is limited (PSNR ≈ 13.2 dB, SSIM ≈ 0.5), as the sparse measurements provide insufficient features for the attention mechanism to resolve. In stark contrast, the high-resolution Human and Horse dataset achieves near-complete reconstruction at N=25% (PSNR ≈ 20 dB, SSIM ≈ 0.57), underscoring the model’s strength in leveraging complex structural information. This dichotomy mirrors the principle that biological perception is most robust when sensory input is information-rich, highlighting that our bio-inspired model’s superiority is most pronounced in complex, real-world scenarios.

## 4. Discussion

The comprehensive results presented in this study demonstrate a clear performance difference between simple and complex datasets. For the structurally rich Human-Horse dataset, the self-attention mechanism effectively leverages the abundant global context, achieving robust reconstruction (PSNR ≥ 23.5 dB, SSIM ≥ 0.7) even at a low sampling ratio (N≤50%) and under high scattering. Conversely, the MNIST dataset, with its low resolution and simple shapes, provides fewer robust features for the attention mechanism to resolve, leading to poorer performance under aggressive undersampling (N≤50%) or extreme scattering. This indicates that the advantage of our bio-inspired approach is most pronounced for complex, real-world scenes, mirroring biological vision which excels in information-rich environments. The methodology positively affects sampling ratios by enabling high-fidelity reconstruction with fewer measurements, as the global context allows it to infer missing information. Self-attention-based GI model achieves robust high-fidelity reconstruction across a wide spectrum of foggy conditions, characterized by scattering coefficients from 0.1 dB/m to 5.0 dB/m and sampling ratios from 10% to 100%. This discussion contextualizes these findings by comparing our model’s performance against state-of-the-art methodologies, elucidating its unique contributions and inherent limitations for computational imaging in remote sensing. However, current limitation of our work is, the model trained and evaluated using a fixed basis of speckle illumination patterns and synthetic fog model based on atmospheric physics (Equations (13)–(16)). While this model is widely used in computational imaging literature [17,18] and provides a controlled environment for testing, its absolute fidelity to real-world fog conditions is a subject for further validation.

**Figure 9:**
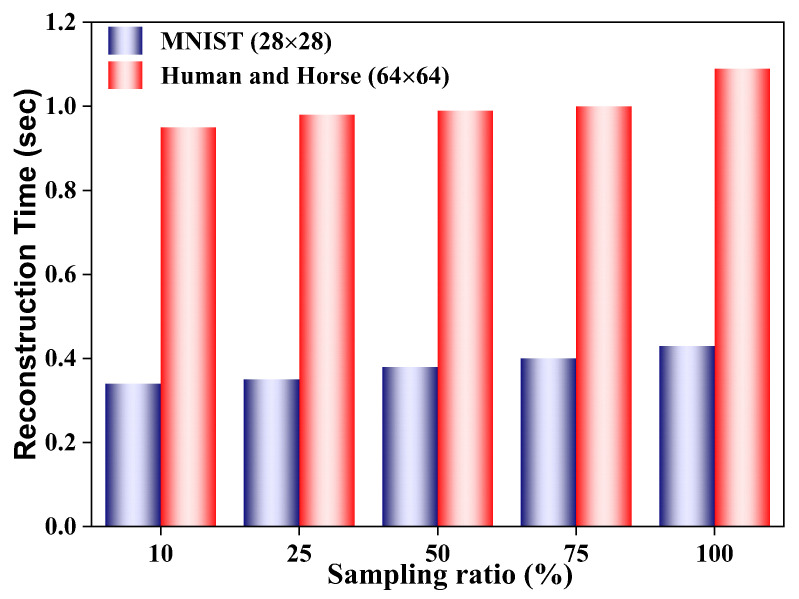
Average reconstruction time (sec) for MNIST (28×28) and Human and Horse (64×64) resolutions under varying sampling ratios at fog density β=21.71dB and Gaussian noise level 10dB. Higher resolution and increased sampling ratios result in longer reconstruction times.

**Comparison with Traditional and CNN-based GI:** The proposed model’s ability to maintain reconstruction quality under high scattering and low sampling distinguishes it fundamentally from traditional GI approaches [19]. For instance, compressive sensing GI [20] achieves reasonable reconstruction with reduced measurements but relies on sparse representations that fail under significant scattering-induced noise. Similarly, while CNN-based methods [21] improve upon traditional techniques, their localized receptive fields limit their ability to integrate global context, leading to pronounced degradation at low sampling ratios (N≤25%). In contrast, the self-attention mechanism of our model, inspired by the brain’s capacity for global feature integration, leverages long-range dependencies within the measurement data. This enables superior performance in high-scattering environments, as evidenced by the high PSNR (≈24.5 dB) and SSIM (≈0.8) achieved for the Human and Horse datasets at β=3.0 dB/m (Figure 7). The primary trade-off in our methodology is the increased computational complexity of the self-attention mechanism compared to simpler CNN architectures. This is a known cost for gaining the ability to model global context. However, as quantified in Table 2 and Figure 9, this cost is manageable and does not preclude real-time application.

**Advancements Over Contemporary GI in Scattering Media:** Recent work by Jiang et al. [22] on GI in atmospheric turbulence achieved improved reconstruction through adaptive correlation but reported lower PSNR values (≈15 dB) under high scattering compared to our model. Our approach demonstrates that a dedicated, learnable attention mechanism is more effective than post-hoc correlation adjustments in mitigating the severe effects of fog. Furthermore, while transformer-based models such as TransUNet [23] excel in domains such as medical image segmentation, their direct application to GI reconstruction in fog remains unexplored and is likely suboptimal because of their task-agnostic design. Our architecture is specifically tailored for the GI reconstruction task, optimizing the self-attention mechanism to directly address scattering-induced degradation, which is confirmed by its higher PSNR and SSIM across varying scattering coefficients (Figure 6c and Figure 7c). The limitation of existing GI methods in scattering media are shown in Table 3.

**The Bio-Inspired Advantage:** The core of the robustness of our model stems from its bio-inspired design. The self-attention mechanism functionally mimics the selective context integration properties of biological visual systems. This allows the model to prioritize salient features and suppress noise, which is particularly advantageous when reconstructing complex scenes from highly degraded or undersampled data. This principled approach moves beyond mere architectural choice and establishes a new paradigm for bio-inspired computational imaging, offering a significant step toward robust remote sensing under real-world adverse conditions.

## 5. Conclusions

This study has presented a transformative, bio-inspired computational imaging framework that significantly advances the robustness of GI for remote sensing in foggy conditions. By integrating a self-attention mechanism explicitly modeled after the brain’s capacity for selective perceptual processing, the proposed model effectively captures both local details and long-range contextual dependencies within highly compressed, one-dimensional bucket signals. This neural-inspired approach enables high-fidelity image reconstruction across a wide spectrum of scattering coefficients (β∈[0.1dB/m,5.0dB/m]) and sampling ratios (N∈[10%,100%]). The main results confirm the following: (1) Our model achieves high-fidelity reconstruction (PSNR 25 dB, SSIM 0.8) under severe scattering (β≤3.0dB/m) and moderate sampling (N = 50%). (2) It outperforms conventional and CNN-based GI methods, particularly in global structure recovery. (3) An ablation study confirms the self-attention module achieved high quality reconstruction. (4) The framework is computationally efficient for real-time use.

The proposed bio-inspired GI framework can be directly integrated with active remote sensing systems such as LIDAR or airborne surveillance platforms. By replacing their conventional reconstruction algorithms with our AMFR-GI model, these systems could achieve significantly enhanced perception in fog, haze, and other obscurants. For real-world deployment, the next step involves training the model on large-scale, real paired data collected from these active systems operating in diverse weather conditions. These advancements position our bio-inspired computational imaging framework as a powerful tool for critical remote sensing applications, including environmental monitoring, security surveillance, autonomous navigation and defense operations under adverse atmospheric conditions.

Current limitations, such as the model’s reliance on a fixed illumination basis and the use of synthetic training data, highlight clear avenues for future work. Exploring hybrid architectures that combine the global context of self-attention with the spatial efficiency of lightweight convolutional layers promises to further optimize this paradigm. Extending the model to be pattern-agnostic and validating it with real-world GI data will be essential to bridge the simulation-to-reality gap. Such developments will continue to harness the principles of biological vision, ultimately creating computational imaging systems that see through obscurants with unprecedented resilience and efficiency.

## Figures and Tables

**Table 1:** Quantitative Comparative analysis under scattering conditions.

Techniques	CGI	CSGI	CNN-CGI	GAN-CGI	Our Model
PSNR	11.70	13.73	14.47	16.01	17.09
SSIM	0.03	0.055	0.21	0.38	0.57

**Table 2:** Computational performance and reconstruction quality across different datasets.

Dataset	Inference Time (s)	Memory Usage (GB)	PSNR/SSIM
			(N=25%)
Human and Horse (64×64)	0.12	2.8	20 dB/0.57
MNIST (28×28)	0.08	1.9	13.2 dB/0.55

**Table 3:** Limitations of existing GI reconstruction methods in scattering media.

Method	Principle	Limitation in Scattering Media
CGI [24]	Linear correlation of patterns and bucket signals.	Fails when noise dominates signal; requires high sampling.
CNN-based GI [25]	Learns nonlinear mapping with local filters.	Limited receptive field struggles with global dependencies needed for scattering correction.
Standard Transformers [26]	Global context via self-attention.	Prohibitive computational cost for high-resolution or real-time GI.
Our model (AMFR-GI)	Bio-inspired, efficient self-attention.	Addresses above by balancing global context with computational efficiency.

## Data Availability

Data underlying the results presented in this paper are not publicly available at this time but may be obtained from the authors upon reasonable request.
